# Low‐Complexity Robotic Device for Magnetic Resonance Imaging‐Guided Needle Biopsy

**DOI:** 10.1002/rcs.70121

**Published:** 2025-11-07

**Authors:** Anastasia Antoniou, Nikolas Evripidou, Leonidas Georgiou, Antreas Chrysanthou, Antonis Christofi, Jufeng Zhao, Liyang Yu, Wenjun Li, Christakis Damianou

**Affiliations:** ^1^ Department of Electrical Engineering, Computer Engineering, and Informatics Cyprus University of Technology Limassol Cyprus; ^2^ Diagnostic and Interventional Radiology Department German Medical Institute Limassol Cyprus; ^3^ Department of Electronics and Information Engineering Hangzhou Dianzi University Hangzhou China

**Keywords:** abdominal, agar phantom, biopsy, MRI, robotic device

## Abstract

**Background:**

This work presents a low‐complexity robotic device for needle‐to‐target alignment designed to streamline Magnetic Resonance Imaging (MRI)‐guided biopsy procedures while maintaining physician control over insertion.

**Methods:**

The robotic device was designed with a rigid frame incorporating two linear degrees of freedom for the in‐plane alignment of a needle guide with a predefined target. Trajectory planning and automated motion execution, including image transfer and sequence triggering, were managed through a custom MRI‐integrated software interface. Preliminary evaluation of alignment accuracy was performed in an agar phantom containing 5 and 10 mm tumour mimics.

**Results:**

In phantom experiments, the system consistently achieved submillimeter alignment accuracy for both the 5‐mm and 10‐mm tumour models across all trials, without any operational failures.

**Conclusions:**

These preliminary findings demonstrate the feasibility of the proposed robotic device for semiautomated MRI‐guided abdominal biopsy, pending further extensive preclinical testing.

## 1 Introduction

Accurate image‐guided abdominal biopsy plays a critical role in the diagnosis and characterisation of lesions in abdominal organs such as the liver and kidneys, particularly when targeting small, deep‐seated, or poorly visualised structures [1]. Among imaging modalities, Magnetic Resonance Imaging (MRI) offers superior soft tissue contrast, multiplanar imaging capabilities, and high sensitivity for detecting abdominal lesions, making it particularly beneficial in complex cases [2]. Nonetheless, performing manual procedures within closed‐bore scanners poses significant technical challenges, including restricted patient access and extended procedure times [3, 4], which have driven efforts towards robotically assisted device solutions to improve precision and procedural efficiency.

Robotic systems have been traditionally developed to improve the accuracy of Ultrasound (US)‐guided needle insertions in percutaneous procedures, streamlining the process and minimising reliance on operator expertise, as demonstrated by numerous phantom‐based studies [5, 6, 7, 8]. Expanding beyond US‐guided systems, robots for CT‐guided needle insertions are available in different configurations, including patient‐mounted [9], table‐mounted [10], and floor‐mounted [11] systems.

Robotic assistance has also proven to be a valuable asset in MRI‐guided interventions [12], enabling automated intra‐procedural adjustments to instrument placement and enhancing overall workflow efficiency. Nevertheless, despite ongoing advancements in the field, robot‐assisted MRI‐guided biopsy has not reached the same level of implementation as US‐ and CT‐guided procedures. It still faces significant challenges, particularly in ensuring MRI compatibility of materials, maintaining functionality without interfering with imaging, and achieving precise motion control through carefully selected actuators and feedback systems [13]. Among these, another key constraint is the limited space within the MRI bore.

In preclinical investigations, He et al. [14] developed a body‐mounted robotic system that employs soft, fluid‐driven actuators for semi‐automated percutaneous needle procedures. The system offers two degrees of freedom (DOF) for adjusting the needle guide, allowing fine automated pitch and manual yaw movements around the surgeon‐defined insertion point. However, the accuracy test only assessed intrinsic targeting, excluding MRI factors such as image distortion and resolution limitations that could affect real‐world performance.

INNOMOTION pioneered in the field being the first MRI‐compatible robotic platform for percutaneous interventions under CT and MRI guidance to be CE‐marked for clinical use. The second generation of this servo‐pneumatically driven system expanded its functionality with a table‐mounted 260° arc supporting a six‐DOF pneumatically driven robotic arm that serves as the end effector [10, 12, 15] with the physician manually inserting the needle. The primary limitations of this system include the large size of the control unit, which may limit its applicability in constrained MRI gantry spaces, and its reduced flexibility in selecting entry points and complexity of multiple DOF.

Motivated by these limitations, this study introduces a practical and compact system for robotic‐assisted abdominal biopsy in the MRI setting. The system features a body‐mounted design with two DOFs, prioritising simplicity and ease of integration. It enables fully automated needle positioning for insertion, with the doctor maintaining control over the insertion process and preserving the experience of haptic feedback. Preliminary validation was performed in a tumour phantom model under controlled conditions in a 3T clinical MRI scanner.

## 2 Materials and Methods

This study did not involve human participants or animals and was conducted entirely using a phantom model. Therefore, ethical approval and informed consent were not required.

### 2.1 Biopsy Needle Positioning Device

**FIGURE 1:**
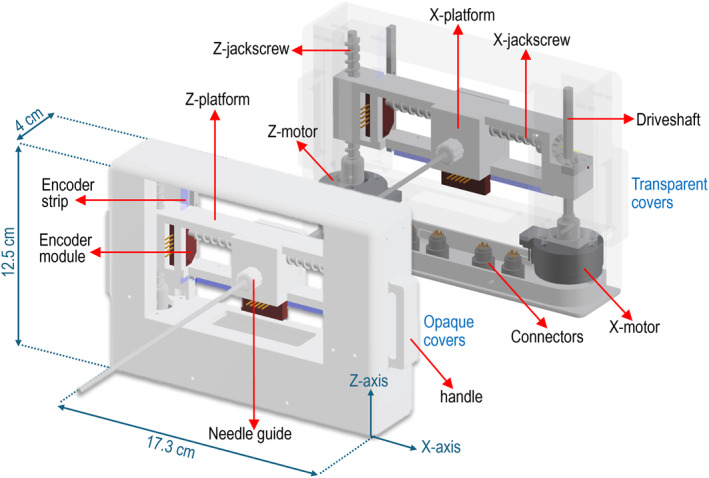
CAD model of the positioning device showing the key components.

The Computer‐Aided Design (CAD) model of the developed needle positioning device is presented in Figure 1 with its various components labelled. The device features two piezoelectrically actuated motion stages, namely the Z‐stage and the X‐stage, for in‐plane positioning of the needle guide along the orthogonal *Z* and *X* axes.

The structure consists of a fixed frame incorporating two piezoelectric motors at the left and right sides of its bottom base, referred to as the X‐motor and Z‐motor (USR30‐S3N, Shinsei Kogyo Corp., Tokyo, Japan). These motors are responsible for actuating a rotational hexagonal driveshaft and the *Z*‐axis jackscrew, respectively, and have demonstrated intrinsic motion accuracy well below 1 mm in our previous robotic platforms [16, 17]. The Z‐stage features a simple design, where the Z‐jackscrew passes through a dedicated channel within the Z‐platform, translating rotational motion into linear displacement along the *Z*‐axis with a motion range of 40 mm. Mounted on the Z‐platform is the X‐platform, which translates along the *X*‐axis through actuation of the corresponding X‐jackscrew, with the Z‐platform frame serving as the guiding structure. The rotational motion of the X‐motor, applied around the *Z*‐axis via a hexagonal driveshaft, is redirected 90° to the *X*‐axis using a bevel gear mechanism. Specifically, a pair of bevel gears is mounted on the driveshaft: the primary (drive) bevel gear rotates when the driveshaft turns, and this gear meshes with a secondary bevel gear oriented orthogonally. This arrangement transfers rotational motion to the X‐stage jackscrew, providing a total motion range of 65 mm. Both platforms are integrated with optical encoder modules that interact with encoder strips aligned along the respective axes (EM1‐0‐500‐I, US Digital, Vancouver, WA, USA), providing real‐time positional feedback.

The needle guide, integrated into the X‐platform, is rigidly fixed perpendicular to the guide frame, thereby constraining insertions to a perpendicular trajectory relative to the phantom/skin surface. To provide flexibility in access, the guide can be translated linearly along the two orthogonal axes within the frame, while the entire frame can be repositioned circumferentially around the body using a dedicated adjustable‐size belt, thereby enabling targeting from multiple surface entry points.

**FIGURE 2:**
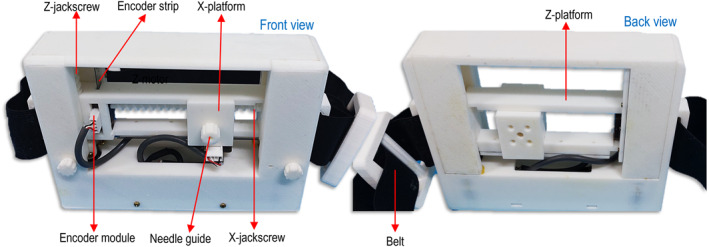
Photographs of the 3D‐printed device with arrows indicating the main visible components.

To ensure MRI compatibility, all structural components of the device were 3D printed from Polylactic acid (PLA) plastic (Raise3D E2 FDM printer, Raise3D, CA, USA) with fastening elements like screws made of brass to prevent magnetic artefacts. Accordingly, mechatronic components (motors and encoders) were selected due to their compatibility with MRI environments and minimal imaging interference when properly positioned [18]. Figure 2 shows front and back views of the 3D‐printed device attached to the belt.

### 2.2 Concept and Workflow of Image‐Guided Needle Positioning

The core principle of the proposed biopsy device is to spatially register a water‐filled syringe (6‐mm diameter) placed at the needle guide with the target using conventional MRI sequences, particularly T2‐Weighted (T2‐W) imaging, which enhances syringe visualisation as a hyperintense marker. Connected to the MRI platform via Access‐I functionality, a dedicated software enables real‐time transfer of imaging data and triggers image acquisition directly, streamlining the workflow and supporting precise image‐guided intervention.

The first step in the targeting workflow is tumour localisation. Quick localiser scans are acquired in three planes (axial, sagittal, and coronal) to identify the approximate tumour position within the phantom or anatomy. The biopsy device is then manually adjusted by the operator to place the syringe marker roughly within the region of interest identified on the scout images.

Next, the operator plans the alignment trajectory using the planning software from outside the MRI room and remains there until accurate needle‐guide alignment is confirmed. The positioning device is initially set to its home position, with the needle guide located at the lower‐left corner of the workspace (*X*, *Z* = 0). Two parallel MRI slices are then acquired: one at the syringe level and one at the target level. The relevant imaging plane is selected to be perpendicular to the intended needle trajectory, defined as the line connecting the axis of the needle guide with the centre of the target. These slices are then fused in the dedicated software to provide in‐plane visualisation of both elements. The operator manually identifies the syringe centre and the target point (a single point within the tumour, typically its geometric centre), which serve as references for trajectory calculation. The coordinate differences $\left(\Delta_{x},\Delta_{z}\right)=\left(x_{t}-x_{g},z_{t}-z_{g}\right)$ are automatically computed and used to command sequential translations of the robot along the *X*‐ and *Z*‐axes, with $\left(x_{t},z_{t}\right)$ and $\left(x_{g},z_{g}\right)$ denoting the coordinates of the target and needle guide centres, respectively.

To confirm accurate in‐plane (lateral) alignment, new parallel scans are acquired and fused, and deviations are recalculated separately along the *X*‐ and *Z*‐axes $\left(\Delta_{x},\Delta_{z}\right)$. An error compensation strategy is implemented by iteratively repeating imaging and overlaying: if the measured deviation exceeds a predefined tolerance T, the needle guide is re‐adjusted, and the process is repeated until both |Δx| and |Δz|≤T. T is defined relative to the imaging resolution (≈1 pixel), which in our implementation corresponds to 0.5 mm. Alignment is accepted only when both |Δx| and |Δz| are within tolerance. Importantly, axial views are used supplementary to provide an orthogonal assessment of angular alignment; that is, ensuring that the planned needle trajectory passes through the centre of the tumour mimic.

**FIGURE 3:**
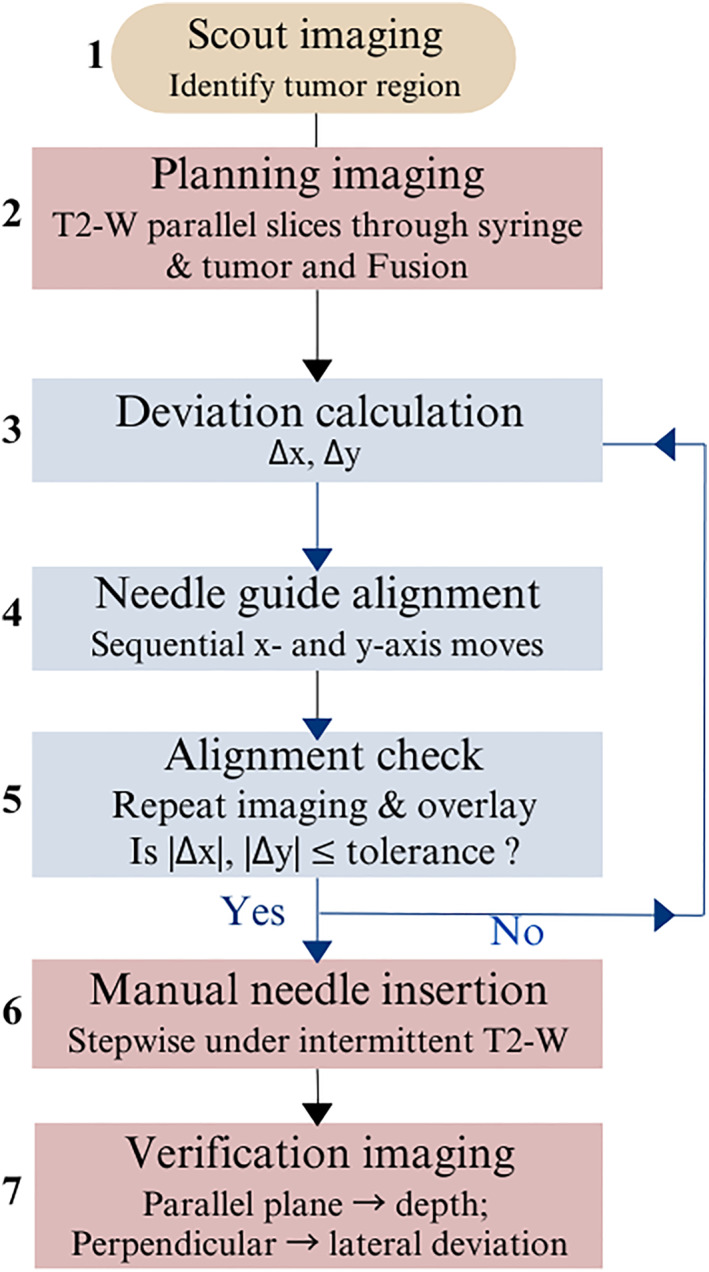
Workflow of the MRI‐guided alignment and insertion procedure. $\Delta_{x},\Delta_{z}$ indicate translational deviations in *X* and *Z*‐axes, respectively.

Once alignment is confirmed, the operator enters the MRI room and performs manual needle insertion in a stepwise manner under intermittent T2‐W imaging in multiple orthogonal planes to monitor needle trajectory and depth relative to the target. A mechanical stop integrated into the needle defines the maximum insertion depth. Depth deviation is assessed from imaging planes parallel to the needle axis, whereas lateral displacement is measured from (fused) perpendicular planes. Although this step was not performed in the present study (since needle tip deviation falls outside the scope of device performance evaluation), the described imaging strategy reflects the expected clinical workflow and will be relevant for future studies assessing needle behaviour during insertion. The complete workflow is summarised in Figure 3.

### 2.3 Proof of Concept in Biopsy Phantom

A tumour‐bearing gel phantom was developed to simulate soft tissue in MRI‐guided needle interventions. Spherical tumour mimics were prepared using a mixture of 6% weight/volume (w/v) agar (Merck KGaA, Darmstadt, Germany) and 6% w/v silicon dioxide (Sigma‐Aldrich, Missouri, USA) in water, measuring 5 and 10 mm in diameter. These tumour mimics were embedded at different depths within a background matrix composed of 6% w/v agar representing healthy tissue, which was formed into a rectangular block. Agar was selected as the base material due to its proven ability to provide tissue‐like signal characteristics in MRI, while also offering mechanical properties for a near‐realistic tactile response during needle insertion [19].

**FIGURE 4:**
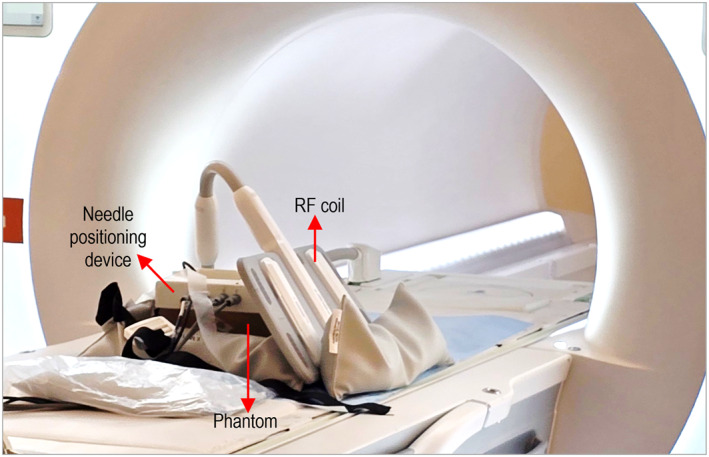
Setup configuration for phantom‐based biopsy trials in the MRI setting.

Phantom trials were performed in a clinical MRI environment (3T Magnetom Vida, Siemens Healthineers, Erlangen, Germany) to assess tumour‐targeting capabilities. The phantom was positioned on the scanner table, and the needle positioning device was securely mounted onto it. A flexible radiofrequency (RF) coil (Ultraflex 18‐channel small coil, Siemens Healthineers) was positioned in two distinct configurations: above the positioning device and laterally alongside the phantom. The lateral coil placement is illustrated in Figure 4. In this configuration, the needle trajectory towards the two embedded tumour mimics was vertical along the posterior–anterior axis.

Trajectory planning and needle‐guide alignment followed the procedure described in Section 2.2 and were repeated five times for each of the two tumour mimics, after which the operator proceeded with insertion following the final alignment attempt. A 3‐mm plastic needle (0.5‐mm tip) was used to avoid imaging artefacts and isolate evaluation of the device itself. It was gradually advanced by a team member in up to four steps with MRI verification at each stage. The total insertion depth was predetermined based on the target location, and depth control was facilitated by external markings on the plastic needle. For all procedural steps, T2‐W imaging, providing high contrast between the agar–silica tumour mimics and the surrounding agar matrix, was performed using either a conventional Turbo Spin Echo (TSE) sequence or a BLADE sequence, selected according to the imaging requirements at each stage of the procedure.

Coronal imaging for planning purposes was found to be more prone to susceptibility artefacts and was therefore performed using a BLADE sequence, which effectively preserved image quality without significant distortion, likely by reducing localised signal inconsistencies introduced by the robotic device compared to the conventional sequence. The parameters used for this sequence were: Repetition time (TR) = 2500 ms, Echo time (TE) = 98 ms, Flip Angle (FA) = 80°, Echo train length (ETL) = 50, Pixel bandwidth (pBW) = 1116 Hz/Pixel, Slice thickness (ST) = 4 mm, Pixel spacing = 1.25 mm, Field of view (FOV) = 400 × 400 mm^2^, and Number of averages (NEX) = 1. BLADE imaging was repeated after movement to evaluate alignment in both coronal and axial planes.

For axial imaging during stepwise needle insertion, an optimised TSE sequence was employed to balance high spatial resolution; necessary for precise visualisation and tracking of the small‐diameter needle with a reduced acquisition time. Specifically, the sequence parameters included TR = 2200 ms, TE = 88 ms, FA = 150°, ETL = 17, pBW = 238 Hz/pixel, ST = 4 mm, FOV = 300 × 245 mm^2^, pixel spacing of 0.7 mm, and NEX = 1, resulting in an acquisition time of approximately 10 s.

## 3 Results

**FIGURE 5:**
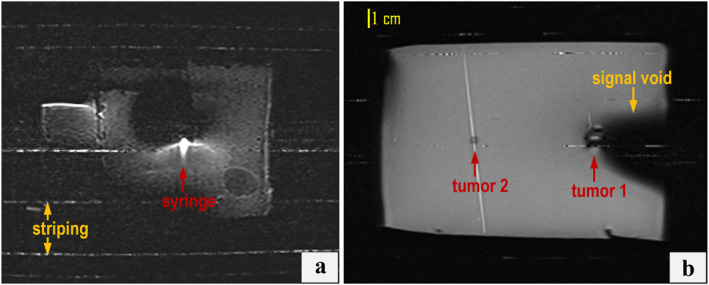
T2‐W TSE images at the level of the (a) syringe and (b) phantom, with the coil placed directly above the phantom‐device setup, showing pronounced interference artefacts.

Positioning the coil above the phantom‐device setup resulted in severe artefacts across both employed sequences. Figure 5 shows examples of compromised T2‐W TSE images at the level of the syringe and tumour mimics within the phantom, displaying prominent artefacts such as signal voids, image distortion, and striping. Note that the syringe was distorted significantly, and its exact position could not be reliably determined, thereby preventing accurate estimation of the in‐plane deviation. As this configuration amplified magnetic susceptibility effects and electromagnetic interference from the device's components, especially around the water‐filled syringe, subsequent scans employed exclusively lateral coil positioning.

**FIGURE 6:**
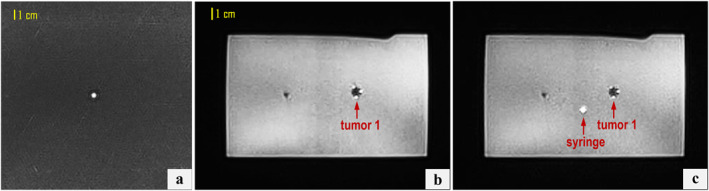
Coronal T2‐W BLADE images acquired prior to needle guide positioning at the levels of (a) the syringe and (b) the 10‐mm tumour mimic, and (c) a fused image of both illustrating the in‐plane deviation between the two.

**FIGURE 7:**
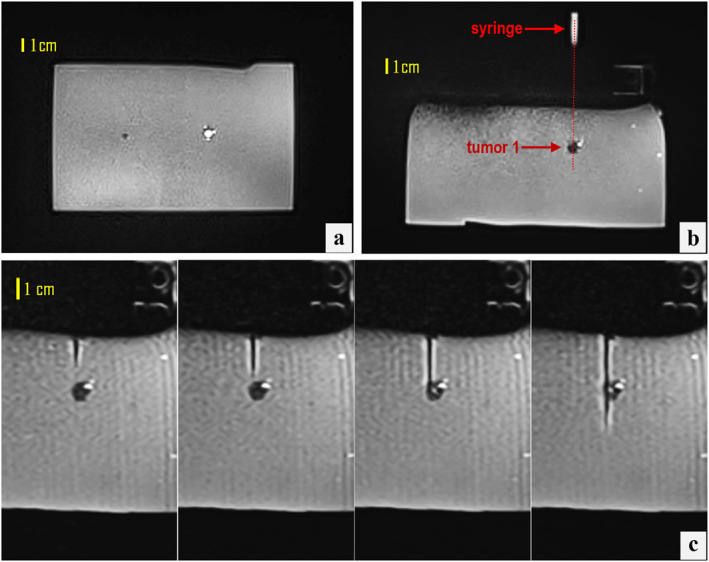
(a) Fused coronal T2‐W BLADE images post‐movement showing the syringe axis intersecting the 10‐mm tumour mimic. (b) Corresponding axial image confirming the angular alignment of the needle guide trajectory with the tumour centre. (c) T2‐W TSE axial images acquired during stepwise needle insertion towards the 10‐mm tumour mimic.

Figure 6 displays indicative T2‐W BLADE images from the planning stage of targeting the 10‐mm tumour mimic, while Figure 7 presents relevant outcomes, particularly the confirmation of needle alignment in both the coronal and axial planes (Figure 7a,b, respectively) as well as the stepwise needle insertion process (Figure 7c) visualised under T2‐W TSE imaging. Note that the plastic needle appeared as a hypointense shaft artefact, with the distal end identifiable at the artefact's termination. In all attempts (*n* = 5), alignment with the tumour centre met the predefined tolerance of one pixel (0.5 mm). This was achieved within a maximum of two adjustment iterations per case, confirming consistent submillimeter alignment accuracy. The needle successfully reached the tumour in all targeting attempts, yielding a 100% puncture success rate, with off‐centre punctures occurring in approximately 30% of cases, typically reflecting operator technique. Figure 7c illustrates an extreme example, where the puncture occurred at the tumour circumference rather than the centre.

Notably, the incorporation of 6% w/v silica increased the stiffness of the tumour mimics compared to the surrounding agar, simulating the firmer consistency of pathological tissue, which the operator could perceive during needle insertion. Furthermore, the selected silica amount sufficiently reduced signal intensity, allowing clear delineation of tumour regions and precise needle targeting with the employed sequences, while also enhancing high‐contrast visualisation of the syringe overlaid on the target tumour in the fused BLADE images.

**FIGURE 8:**
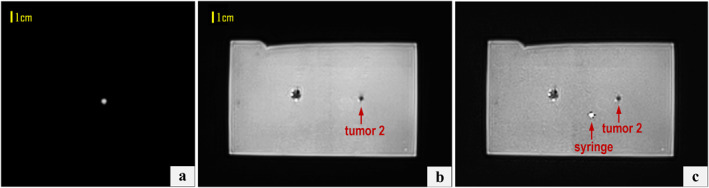
Coronal T2‐W BLADE images acquired prior to needle guide positioning at the levels of (a) the syringe and (b) the 5‐mm tumour mimic, and (c) a fused image of both illustrating the in‐plane deviation between the two.

**FIGURE 9:**
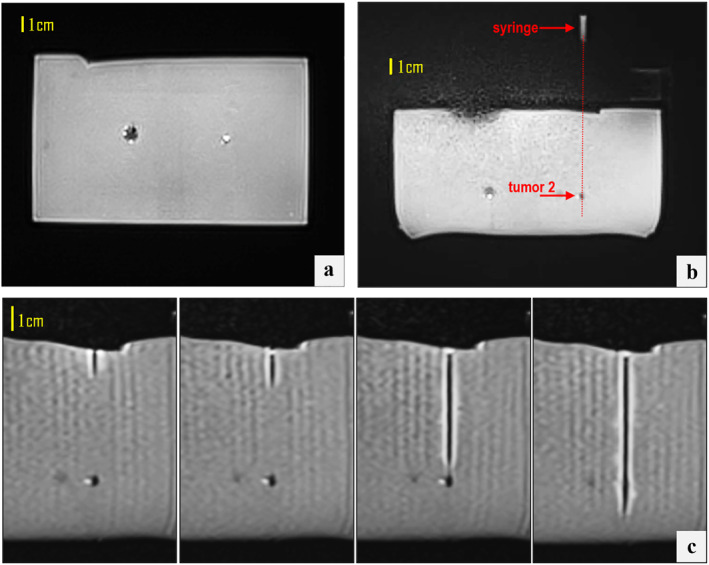
(a) Fused coronal T2‐W BLADE images post‐movement showing the syringe axis intersecting the 5‐mm tumour mimic. (b) Corresponding axial image confirming the angular alignment of the needle guide trajectory with the tumour centre. (c) T2‐W TSE axial images acquired during stepwise needle insertion towards the 5‐mm tumour mimic.

Corresponding results for the smaller 5‐mm tumour, from the planning phase to needle guide alignment and insertion, are shown in Figures 8 and 9, respectively. Remarkably, post‐alignment superimposed coronal slices reveal the syringe overlapping the small tumour due to their similar sizes. Mean duration of the total robot procedure (device positioning, image acquisition and transfer, trajectory planning and needle guide‐to‐target alignment) was 15 ± 2 min per procedure.

## 4 Discussion

This study introduced a simplified, MRI‐compatible robotic device for needle positioning, along with a corresponding workflow, aimed at improving the precision and efficiency of traditional MRI‐guided abdominal biopsies. The approach aims to reduce the complexity and challenges of integrating advanced robotic systems with MRI, thereby supporting the adoption of MRI‐guided, robot‐assisted biopsy in routine clinical practice. Unlike the commercially available Innomotion system [15], which is large and complex and features multiple DOFs, our device is compact, with a simplified mechanism that focuses specifically on precise needle guide alignment while allowing the physician to maintain control over needle insertion. Additionally, the device's piezoelectric stages provide direct linear motion with reduced mechanical complexity compared to the fluid‐driven actuator system reported in earlier work [14], which relies on deformable structures and complex feedback control.

The developed device consists of two piezoelectrically actuated linear motion stages housed within a compact rectangular frame and is remotely controlled via dedicated software. Its integration efficiency within the MRI environment and operational workflow were evaluated using a phantom‐based setup. Coil positioning relative to the device was critical in minimising image artefacts. Two configurations were evaluated: one with the coil placed above the phantom‐device setup, and the other laterally. Lateral positioning increased the distance between the coil and the device's piezoelectric motors and cables, while also altering their relative orientation. This adjustment reduced overlap between the coil's RF field and device noise, ensuring clear signal reception for accurate trajectory planning and real‐time MRI monitoring with minimal distortion and no mechanical interference.

The methodology employed for MRI‐guided automated needle guide‐to‐target alignment successfully demonstrated a proof of concept. The spatial relationship between the imaging space and the robotic device was established using a water‐filled syringe, which functioned as a marker in T2‐W imaging. Parallel cross‐sectional images through the syringe and target centre, oriented perpendicular to their connecting axis, provided a straightforward and efficient means of estimating in‐plane deviation within the software.

Assessment of guide alignment accuracy relative to the target was prioritised in this proof‐of‐concept study, as this represents the most relevant performance metric for the robot's intended clinical use, that is, aligning the needle guide with the planned trajectory, while needle insertion is performed manually by the physician. The trials consistently achieved alignment within the predefined 0.5 mm tolerance, highlighting the reliability of the alignment strategy. It should be noted that this level of accuracy reflects the device's motion performance within the MRI environment, accounting for MRI‐related effects including inherent image distortion and resolution limitations, contingent on precise spatial registration within the MRI coordinate system. This suggests that device actuation was not impaired by magnetic interference and that software communication was reliable.

Once MRI‐confirmed alignment has been achieved, needle insertion accuracy (terminal placement precision) does not represent device performance and is largely influenced by operator‐dependent factors such as hand stability, control of insertion angle, overall experience, and needle flexibility. In clinical practice, these factors are expected to be further affected by needle–tissue interactions and amplification of small errors over greater insertion depths. For this reason, puncture accuracy was assessed qualitatively based on the success rate of target puncture and any visually observed deviation from the centre. Accordingly, insertion‐related parameters such as angular deviation and procedural insertion time were not analysed, as they primarily reflect manual operator performance rather than device functionality.

Several MRI‐compatible robotic systems have been developed to facilitate biopsy procedures. The Stormram‐4 system for MRI‐guided breast biopsy achieved 3D terminal accuracy (tip‐to‐target) close to 2 mm under imaging conditions [20]. Similarly, phantom experiments with a long‐bone biopsy robot reported a 3D targeting error of 1.39 ± 0.40 mm [21]. Other systems have been developed as general‐purpose MRI‐compatible platforms for needle‐based interventions, with biopsy as one of several potential applications. For instance, a body‐mounted MR‐conditional liver robot was proposed by Huang et al. [22], demonstrating a targeting error of 2.6 ± 1.3 mm in free‐space experiments. Moving to in vivo testing, a body‐mounted shoulder arthrography robot evaluated in cadaver studies reported a mean translational terminal error of 2.07 ± 1.22 mm [23]. Accuracy metrics reported for these devices are not directly comparable to ours, as they primarily reflect terminal accuracy after needle insertion, whereas our study specifically evaluates guide‐to‐target alignment accuracy.

INNOMOTION, the only commercialised MRI‐compatible robotic platform for percutaneous interventions, supports a broad range of procedures including biopsy, ablation, and joint injections. Bench testing demonstrated mechanical targeting precision with a mean deviation of < 0.5 mm (maximum < 1.5 mm), while MRI‐guided targeting accuracy was ∼1–2 mm when systematic imaging errors were considered [10]. In this initial evaluation, our system achieved submillimeter alignment accuracy under MRI guidance, showing that even a simplified platform can reach levels of precision comparable to more complex commercial systems.

A limitation of the present prototype is that the needle is constrained to perpendicular insertion relative to the skin surface, which may restrict its applicability in procedures requiring strongly angulated trajectories. Future iterations of the device will address this by incorporating a rotatable or articulated needle guide, enabling controlled angular adjustment of the insertion path without altering the overall frame design or workflow. Furthermore, while phantom experiments are essential for initial testing, they do not fully replicate the complexities of in vivo environments, such as variations in tissue characteristics and patient movement. Therefore, further validation in more realistic phantom models that mimic abdominal anatomy and include harder‐to‐access targets, followed by in vivo studies, is warranted.

In clinical applications, the choice of imaging sequences will vary depending on the target and will be guided by key criteria: minimising susceptibility artefacts, achieving sufficient spatial resolution for accurate visualisation of both the target and the syringe or needle, and maintaining short acquisition times to optimise workflow efficiency. By automatically aligning the needle guide to the target, the device reduces the need for repeated patient repositioning. Automated alignment, combined with the simplicity of the device and workflow, is expected to provide measurable time savings, although the total duration of a complete clinical procedure cannot presently be calculated, as it will remain case‐specific.

## 5 Conclusions

The system is designed to balance automation and physician control, with the robot providing accurate positioning and the physician maintaining haptic feedback and clinical judgement. The design prioritises simplicity and safety to support reliable needle‐guide alignment. In this preliminary assessment, performance was evaluated under controlled phantom conditions using a plastic needle, demonstrating reliable alignment accuracy. As a next step, more realistically shaped phantoms could be used, followed by a smooth transition to in vivo testing in animal models. Clinical translation would require addressing additional factors, including patient motion, physiological variability, and the use of real biopsy needles.

## Author Contributions

Anastasia Antoniou analysed and interpreted the data and wrote the manuscript. Nikolas Evripidou designed the robotic device and prepared the phantom model. Leonidas Georgiou, Antreas Chrysanthou, and Antonis Christofi contributed to the phantom experiments and collection of MRI data. Jufeng Zhao, Liyang Yu, Wenjun Li, and Christakis Damianou contributed to the study's conceptual framework, provided expert guidance on the experimental design, and critically reviewed the manuscript. All authors reviewed and approved the final manuscript.

## Funding

The authors have nothing to report.

## Ethics Statement

The authors have nothing to report.

## Consent

The authors have nothing to report.

## Conflicts of Interest

The authors declare no conflicts of interest.

## Data Availability

The data that support the findings of this study are available on reasonable request from the corresponding author.
